# SDH mutations, as potential predictor of chemotherapy prognosis in small cell lung cancer patients

**DOI:** 10.1007/s12672-023-00685-4

**Published:** 2023-06-05

**Authors:** Ran Zeng, Xiaoyun Fan, Jin Yang, Chen Fang, Jieyi Li, Wei Wen, Jing Liu, Mengchen Lv, Xiangran Feng, XiaoKai Zhao, Hongjie Yu, Yuhuan Zhang, Xianwen Sun, Zhiyao Bao, Jun Zhou, Lei Ni, Xiaofei Wang, Qijian Cheng, Beili Gao, Ziying Gong, Daoyun Zhang, Yuchao Dong, Yi Xiang

**Affiliations:** 1 Department of Pulmonary and Critical Care Medicine, Ruijin Hospital, Shanghai Jiao Tong University School of Medicine, No.197, Rui Jin 2nd Road, Shanghai, 200025 People’s Republic of China; 2 Department of Geriatric Respiratory and Critical Care, The First Affiliated Hospital of Anhui Medical University, Hefei, 230022 China; 3 Department of Respiratory and Critical Care Medicine, Shanghai Changhai Hospital, The First Affiliated Hospital of Second Military Medical University, No.168, Changhai Road, Yangpu District, Shanghai, 200433 People’s Republic of China; 4 Jiaxing Key Laboratory of Precision Medicine and Companion Diagnostics, Jiaxing Yunying Medical Inspection Co., Ltd, Building 5, No.3556 Linggongtang Road, Nanhu District, Jiaxing, 314000 People’s Republic of China; 5 Department of R&D, Zhejiang Yunying Medical Technology Co., Ltd, No.158 Huixin Road, Nanhu District, Jiaxing, 314000 People’s Republic of China; 6 Institute of Respiratory Diseases, Shanghai Jiao Tong University School of Medicine, Shanghai, 200025 China; 7 Shanghai Key Laboratory of Emergency Prevention, Diagnosis and Treatment of Respiratory Infectious Diseases, Ruijin Hospital, Shanghai Jiao Tong University School of Medicine, Shanghai, 200025 China

**Keywords:** SCLC, *SDH* mutations, Chemotherapy prognostic, Random forest, Next generation sequencing

## Abstract

**Purpose:**

Small cell lung cancer (SCLC) is an aggressive and rapidly progressive malignant tumor characterized by a poor prognosis. Chemotherapy remains the primary treatment in clinical practice; however, reliable biomarkers for predicting chemotherapy outcomes are scarce.

**Methods:**

In this study, 78 SCLC patients were stratified into “good” or “poor” prognosis cohorts based on their overall survival (OS) following surgery and chemotherapeutic treatment. Next-generation sequencing was employed to analyze the mutation status of 315 tumorigenesis-associated genes in tumor tissues obtained from the patients. The random forest (RF) method, validated by the support vector machine (SVM), was utilized to identify single nucleotide mutations (SNVs) with predictive power. To verify the prognosis effect of SNVs, samples from the cbioportal database were utilized.

**Results:**

The SVM and RF methods confirmed that 20 genes positively contributed to prognosis prediction, displaying an area under the validation curve with a value of 0.89. In the corresponding OS analysis, all patients with *SDH*, *STAT3* and *PDCD1LG2* mutations were in the poor prognosis cohort (15/15, 100%). Analysis of public databases further confirms that *SDH* mutations are significantly associated with worse OS.

**Conclusion:**

Our results provide a potential stratification of chemotherapy prognosis in SCLC patients, and have certain guiding significance for subsequent precise targeted therapy.

**Supplementary Information:**

The online version contains supplementary material available at 10.1007/s12672-023-00685-4.

## Introduction

Small cell lung cancer (SCLC) is a common yet aggressive carcinoma with an extremely high proliferation rate, accounting for about 15% of cases of the most lethal carcinoma, lung cancer [1]. Due to its extraordinary invasiveness, SCLC is prone to early metastases, leading to generally poor curative outcomes [2]. In contrast to the increasing benefits of precision treatment for non-small cell lung cancer (NSCLC) patients in recent years, the current clinical treatment for SCLC remains inadequate. Concurrent chemoradiotherapy (CRT) is the primary clinical treatment for limited-stage (LS) SCLC, while chemotherapy alone is employed for extensive-stage (ES) SCLC [3]. However, most patients’ responses to CRT or chemotherapy are transient, followed by rapid recurrence and dismal survival rates, with a median survival time of less than 2 years for early patients and about one year for patients with metastases [2]. A small number of SCLC patients exhibit initial resistance to the first-line standard etoposide combined with carboplatin or cisplatin (EC or EP) regimen, resulting in more rapid progression and poor prognosis after treatment. Accurate identification of primary drug resistance, early acquired drug resistance, and patients with poor prognosis remains elusive. Consequently, the majority of inoperable advanced SCLCs are indiscriminately treated with EP or EC regimens, highlighting the urgent clinical need for first-line chemotherapy.

With advancements in technologies such as next-generation sequencing (NGS), molecular profiling of SCLC has made unprecedented progress [4, 5]. These DNA-level studies have provided insights into the genetic variation profile and genetic nature of SCLC [6]. Tumor suppressor genes *TP53* and *RB1* were universally inactivated in SCLC, which was once considered a molecularly homogeneous tumor [7], In recent years, the importance of *MYC* family, *KMT2D*, *PIK3CA*, and other gene mutations has been confirmed in patients, xenograft tumor models, mice, and cell levels. SCLC is characterized by complex and variable gene mutations, high intra-tumoral heterogeneity, and plasticity [3, 8]. With the progress of molecular precision diagnosis and treatment in melanoma, NSCLC, and other carcinoid tumors, the research direction of subtyping SCLC based on molecular gene status to predict therapeutic response and achieve first-line precision treatment has attracted considerable attention from researchers [9, 10]. Different stages of SCLC, surgical intervention, and first-line treatment regimens are crucial prognostic indicators for patients. However, significant differences exist in the efficacy of surgery and standard chemotherapy in patients with the same stage [11]. Previous studies have explored biomarkers in tissue protein expression, blood tumor markers, blood immune cell counts, and biochemical indicators, but their clinical application is limited due to small experimental samples and varying detection platform standards [2]. Currently, clinical practice requires feasible biomarker research to predict the efficacy and clinical outcomes of first-line EP or EC chemotherapy in advanced SCLC to guide precise treatment in clinical practice.

Machine learning methods can process massive, high-dimensional, high-throughput data, identify gene changes with high contributions, and use a small number of gene markers to achieve accurate prognosis prediction [12–14]. In this study, leave-one-out cross-validation (LOOCV) and random forest (RF) methods are employed for data processing [15, 16]. These methods can efficiently apply data and avoid overfitting of subsequent models, making them suitable for studies with precious sample sources, such as SCLC [9].

This study aims to identify reliable biomarkers for predicting the efficacy and clinical outcomes of first-line chemotherapy treatments in advanced SCLC patients. We employed machine learning methods, such as random forest (RF) and support vector machine (SVM) algorithms, to process complex genetic data and accurately predict patient prognosis based on molecular gene status. Our findings have the potential to guide precise treatment decisions in clinical practice, ultimately improving survival outcomes for SCLC patients.

## Patients and methods

### Patient selection

This research was designed as a multi-center retrospective study. Patients diagnosed with SCLC at Ruijin Hospital (Shanghai, China) from January 2013 to December 2020 and Changhai Hospital (Shanghai, China) from January 2018 to December 2020, who met the inclusion criteria, were enrolled in this study (Supplementary methods). Data regarding baseline characteristics, including age at diagnosis, gender, Eastern Cooperative Oncology Group Performance Status (ECOG PS) score, tumor stage, smoking habits, pathologic type, metastases, clinical treatment, and outcomes, were carefully collected for each participant. Tumor evaluation was performed according to the revised Response Evaluation Criteria in Solid Tumors (RECIST) guidelines (version 1.1) [17]. All patients were classified into a two-stage system (limited-stage and extensive-stage) according to the Veterans Administration Lung Cancer Group (VALG) staging method [2, 18]. Overall survival (OS) was defined as the time from diagnosis to death from any cause. Based on phase III clinical studies and meta-analysis data [19–21], patients were assigned to either the “good prognosis” (OS ≥ 10 months) or “poor prognosis” (OS < 10 months) cohort.

### DNA extraction and library construction

Paraffin-embedded tumor tissue specimens were provided by each patient. A pathologist micro-dissected all samples selected for sequencing to confirm regions with > 70% tumor content. Tissue DNA extraction and purification were performed using the human tissue DNA extraction kit (Yunying Medicine, Ltd., Zhejiang, China). DNA concentration and purity were assessed using NanoDrop2000 (Thermo Fisher Scientific, Waltham, MA, USA). Prepared samples were stored at − 20 °C until use. Targeted sequencing strategies were employed in this study, and the library was prepared using the VAHTS Universal DNA library prep kit (Illumina, Carlsbad, CA, USA). Target enrichment was performed using optimized probes (Yunying Medicine, Ltd.) designed to capture exons and some introns, targeting mature transcripts of 315 cancer-related genes. Sequencing was performed using the NextSeq500 platform (Illumina, Carlsbad, CA, USA), with each experimental step strictly following the manufacturer's protocol.

### Next-generation sequencing (NGS)-based assay

FASTQ files were screened using FastQC software (version 0.11.2) and a custom Python script to remove adapter sequences and sequences with a Q score below 30. The Burrows-Wheeler Aligner (BWA, version 0.7.7) was employed to map clean reads to the reference human genome GRCh37/hg19. The resulting BAM files were realigned and recalibrated using GATK3.5, which was also used to detect mutations. To reduce potential polymerase chain reaction bias, duplicate sequences were removed using Picard MarkDuplicates (version 1.35). Single nucleotide variations (SNVs) were identified using VarScan (version 2.3.2), and SNVs meeting the following criteria were retained: allele frequency ≥ 10%, total reads ≥ 100, and changed reads ≥ 50. The Yunying internal germline SNV database (Yunying Medicine, Ltd., Zhejiang, China) was employed to filter germline SNVs, which was built upon the sequencing results of 2,588 samples, and only SNVs with a frequency of more than 10% in individuals were considered [22]. The obtained somatic SNV results were verified using the Integrative Genomics Viewer (IGV, version 2.4.1) to further remove unreliable candidate sites.

### SNV selection using random forest (RF) algorithm

The SNV clustering process was conducted on patient samples and genes using CIMminer software (http://disconver.nci.nih.gov/cimminer). The “randomForest” R package [23] was employed with the ‘important’ option set to true. The RF algorithm was then used to analyze SNV data from all 78 patients, operating in a classification mode. Only SNVs with positive accuracy contributions were selected and subjected to further screening with the RF classifier. Progressive screening continued until only SNVs that boosted classification accuracy remained, and Out-of-Bag (OOB) errors stopped declining. In the RF classification algorithm, 2/3 of the samples were used to build the decision tree, with the rest reserved for validation. The OOB error, an evaluation metric derived from verification, was used to monitor the accuracy of the classifier, with decreasing OOB error implying improved classification accuracy. In the last stages of progressive screening, when a limited number of SNVs were removed and OOB stabilized, SNVs that lowered classification accuracy in certain random cases were considered for further modeling.

### Predictive modeling using support vector machines (SVMs)

We conducted a four-fold cross-validation experiment. The samples were randomly divided into four groups, each with a class distribution that was maintained. Each group was then used as the testing set in turn, with the other groups serving as the training set. The overall performance was reported as the mean and standard deviation of the results from the four SVM models generated through cross-validation. To assess the impact of SNVs with uncertain effects on SVM performance, we repeated the four-fold cross-validation process 1000 times with different random group selections. The subset with the highest mean ROC curve AUC was selected as the final set for further investigation.

### Public database verification

cBioPortal (https://www.cbioportal.org/) was employed for the prognostic ability of SNVs. Briefly, the detailed clinical baseline, gene sequencing and treatment information of 239 patients with SCLC were downloaded, and removed the duplicate samples.

### Bioinformatic analysis

All genes harboring mutations were recorded, and pathway mapping and enrichment analysis were performed using Gene Ontology (GO) and Kyoto Encyclopedia of Genes and Genomes (KEGG) via the R package “clusterProfiler” (v.3.14.3) [24]. Agilent Literature Search (v.3.1.1) [25] in Cytoscape [26] was used to search and generate the gene-regulatory network.

### Statistical analysis

One-way analysis of variance was used to assess the effects of mutations, followed by Fisher’s exact test to independently compare variations between groups, and a Mann–Whitney U test to determine differences in total variations between the two cohorts. Variation burden was analyzed using the Poisson test, and survival analysis was based on the Kaplan–Meier method. All statistical analyses were performed, and odds ratios (ORs) generated with SPSS (v.22.0; IBM Corp., Armonk, NY, USA), with a P < 0.05 considered statistically significant. Correlations (r) were calculated using Kendall’s tau-b or tetrachoric correlation methods. The R package “survival” was used for Kaplan–Meier survival analysis, and other statistical charts were generated by the R package “ggplot2” [27].

## Results

### Demographic and clinical characteristics of included patients

From 2013 to 2020, 78 patients newly diagnosed with SCLC and who received standard first-line etoposide plus carboplatin or cisplatin therapy were enrolled in the study (Fig. S1), including 60 samples from Ruijin Hospital and 18 samples from Changhai Hospital. Slightly more than half of the patients (44, 56.4%) were younger than 65 years old, and the male/female ratio was 73/5. More than three-quarters of patients (59, 75.6%) had been or were smokers. Clinical staging statistics revealed that 51 patients were evaluated as extensive (65.4%) at the time of diagnosis, of which 35 (44.9%) patients had extrapulmonary distant metastasis, and most (73/78) patients were in good basic condition (ECOG score 0–1).

In terms of prognosis, 66 cases of progression after first-line treatment (progression-free survival, PFS event maturity 84.6%) and 46 cases of death events (maturity 59.0%) were observed in the study. The median PFS of enrolled patients was 7.15 months (interquartile range, IQR: 4.13–11.02 months), and the median overall survival (mOS) was 16.52 months (IQR: 10.03–25.32 months). Referring to previous meta-analysis and Phase III clinical trials [20–22], patients were divided into a group of good prognosis (OS ≥ 10 months) and another poor cohort (OS < 10 months) by a cut-off value of 10 months. By this means, 60 patients (76.8%) had a good prognosis and 18 patients (23.2%) had a poor prognosis. The clinical characteristics (age, gender, ECOG PS status, distant metastasis status, smoking status; shown in Table 1 and Table S1) of both prognosis groups are similar, but there are differences in staging: compared with the good cohort, the poor one is more in the extensive stage (P = 0.023). In addition, although statistically insignificant, the proportion of patients with good prognosis receiving chest radiotherapy was higher (61.7% vs 33.3%, P = 0.057).

**Table 1:** Clinical characteristics of SCLC patients receiving first-line platinum containing dual drug chemotherapy

	Total patients	Good prognosis (OS ≥ 10 m)	Poor prognosis (OS < 10 m)	P value
	N(%)	N(%)	N(%)	
Age				
< 65 years old	44(56.4)	33(55.0)	11(61.1)	0.788
≥ 65 years old	34(43.6)	27(45.0)	7(38.9)	
Gender				
Male	73(93.6)	56(93.3)	17(94.4)	1.000
Female	5(6.4)	4(6.7)	1(5.6)	
Staging				
Limited-stage	27(34.6)	25(41.7)	2(11.1)	**0.023**^a*^
Extensive-stage	51(65.4)	35(58.3)	16(88.9)	
Distant metastasis				
No	43(55.1)	35(58.3)	8(44.4)	0.418
Yes	35(44.9)	25(41.7)	10(55.6)	
ECOG PS				
0–1	73(93.6)	58(96.7)	15(83.3)	0.078
2–3	5(6.4)	2(3.3)	3(16.7)	
Smoking				
Never	19(24.4)	17(28.3)	2(11.1)	0.211
Present/past	59(75.6)	43(71.7)	16(88.9)	
Chest radiotherapy				
No	35(44.9)	23(38.3)	12(66.7)	0.057
Yes	43(55.1)	37(61.7)	6(33.3)	
Posterior line immunotherapy				
No	71(91.0)	53(88.3)	18(100)	0.192
Yes	7(9.0)	7(11.7)	0(0)	
Total number of patients	78(100)	60(76.8)	18(23.2)	

*ECOG PS* Eastern Cooperative Oncology Group Performance Status; *m* month

^a^Bold value, statistically significant

*at the level of P < 0.050

### SCLC gene mutation map and random forest screening

**Fig. 1:**
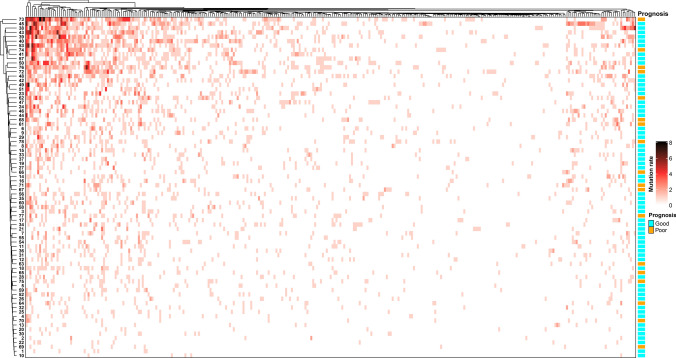
Mutation landscape of SCLC patients receiving platinum-based standard chemotherapy

**Fig. 2:**
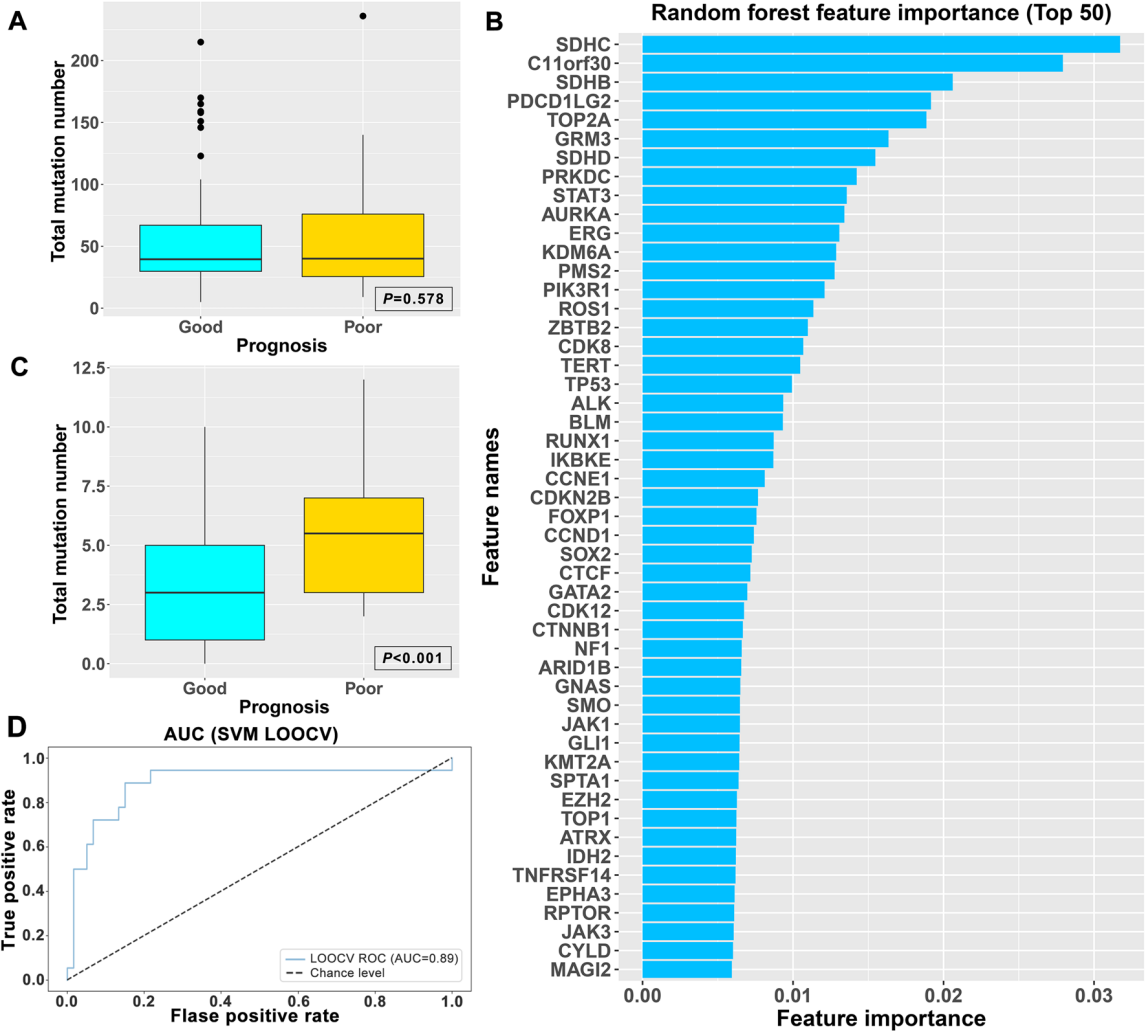
Prognostic-related mutated genes selected by random forest (RF) and support vector machines (SVM). **A** Total number of mutations per person for the two groups of 315 genes, **B** the top 50 genes and RF values for the random forest screening, **C** the total number of mutations per person for the two groups of the top 20 genes, **D** ROC curve of a prognostic model built by SVM-LOOCV

The landscape of patient genotypes was explored using next-generation sequencing (NGS) to investigate the molecular characteristics of SCLC. A panel of 315 genes related to tumorigenesis was selected to identify differences in patient genotypes. Bioinformatic analyses of this panel revealed that the top ten frequently mutated genes were *TP53*, *RB1*, *KMT2C*, *KMT2D*, *LRP1B*, *KMT2A*, *FAT1*, *PRKDC*, *BRCA2*, and *EP300*. The mutations in 78 patients at the gene level were determined and clustered, and there was no significant correlation with the efficacy and prognosis of first-line standard chemotherapy (Fig. 1). Among the 315 genes, there was no significant difference in the total number of mutations per capita between patients with good prognosis and those with poor prognosis (P = 0.578, Fig. 2A).

Contributive SNVs to the prognosis were screened by random forest (RF) analysis, and the top 50 obtained genes are shown in Fig. 2B. The top 10 included *SDHC*, *C11orf30*, *SDHB*, *PDCD1LG2*, *TOP2A*, *GRM3*, *SDHD*, *PRKDC*, *STAT3*, and *AURKA*. The top 20 genes contributing to clinical survival outcomes were included to construct an SVM model. After screening the top 20 determinant SNVs by RF, the total number of mutations per capita in the cohort of patients with poor prognosis was significantly higher than that in the cohort with good prognosis (P < 0.001, Fig. 2C).

### SVM classification model and evaluation

A cross-validation of the SVM-LOOCV algorithm was applied to confirm the positive contribution of the top 20 genes selected by RF. As shown in Fig. 2D, the area under the validation curve (AUC) of SVM-LOOCV for prognosis is 0.89, while the AUCs for staging and distant metastasis are 0.65 and 0.57, respectively, suggesting that the SVM model has good prognostic differentiation ability. We further conducted single-factor and multi-factor survival analyses on the predictive ability of the model (Table S2 and S3) and found that the model grouping is an independent predictor of PFS and OS (PFS: HR = 2.820, 95% CI 1.371–5.801, P = 0.005; OS: HR = 2.512, 95% CI 1.107–5.701, P = 0.028). Therefore, combined with the cross-validation characteristics of SVM-LOOCV and the survival analysis of model grouping, the top 20 genes model possesses a certain ability to predict clinical prognosis.

**Fig. 3:**
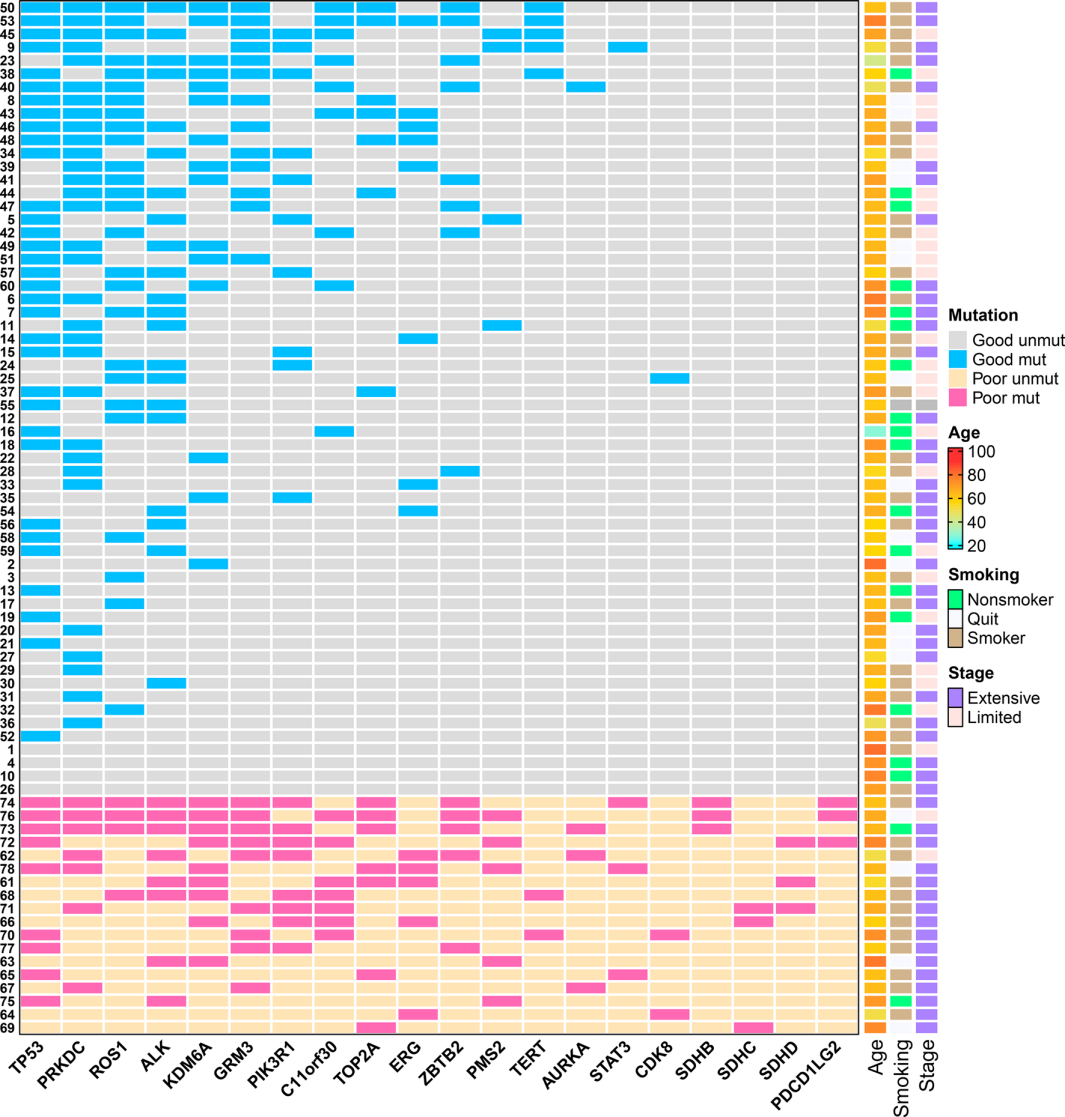
Complex heatmap of the top 20 genes SNV profiling selected through random forest (RF) and their clinical characteristics

The details of the 20 gene mutations are shown in Fig. 3 in the form of a heatmap. Additionally, we observed that despite the low mutation frequency (3.85%) of *SDHC* (3/78), *SDHB* (3/78), and *SDHD* (3/78), all patients with *SDH* family gene mutations belong to the poor prognosis cohort (8/8, 100%). This suggests that the *SDH* family may have a certain correlation with the prognosis of SCLC patients.

### Survival analysis of key mutant genes

To verify the predictive capability of the top 20 genes selected by random forest analysis, we analyzed the distribution of these genes in the two cohorts with good and poor prognosis. The results revealed statistically significant diversity in mutation frequencies for 9 genes (*PIK3R1*, *TOP2A*, *PMS2*, *STAT3*, *AURKA*, *SDHB*, *SDHC*, *SDHD*, *PDCD1LG2*) between the two cohorts (Table S4), with patients carrying these gene mutations tending to have worse prognosis. Interestingly, mutations in the *MYC* family, *KMT2D*, and *PIK3CA*, which are thought to have significant effects on SCLC, were not selected as prognostic features by the random forest model in our cohort. Upon further examination of the distribution of these genes in our cohort, we found that their mutations did not influence patients' chemotherapy outcomes (Table S5).

**Fig. 4:**
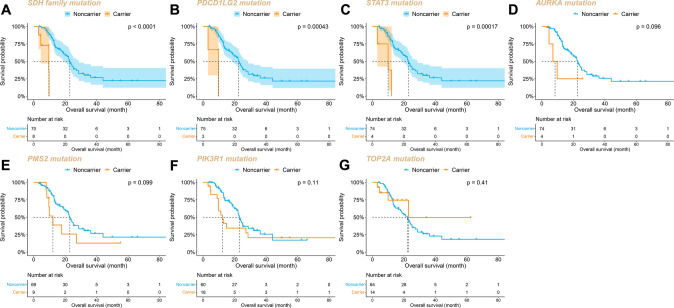
Kaplan Meier curves for OS according to the mutation of **A** *SDH* family, **B** *PDCDLG2*, **C** *STAT3*, **D** *AURKA*, **E** *PMS2*, **F** *PIK3R1*, and **G** *TOP2A*

To further explore the 9 genes with significant Poisson distribution divergence, we conducted a Kaplan–Meier survival analysis for the two prognostic cohorts. Log-rank analysis demonstrated that the OS of patients with *SDHB*/*C*/*D* mutations was shorter than that of non-carriers after receiving first-line standard EC or EP regimen chemotherapy (mOS: 23.0 m vs 9.3 m, P < 0.0001, Fig. 4A). Additionally, the prognosis of patients with *PDCD1LG2* and *STAT3* gene mutations was worse than that of non-carriers (*PDCD1LG2* mOS: 22.6 m vs 9.7 m, P = 0.00043, Fig. 4B; *STAT3* mOS: 23.0 m vs 10.0 m, P = 0.00017, Fig. 4C). Except for *TOP2A*, patients with mutations in *AURKA*, *PMS2*, and *PIK3R1* genes tended to have worse prognosis, although the difference was not statistically significant (Fig. 4D–G).

**Fig. 5:**
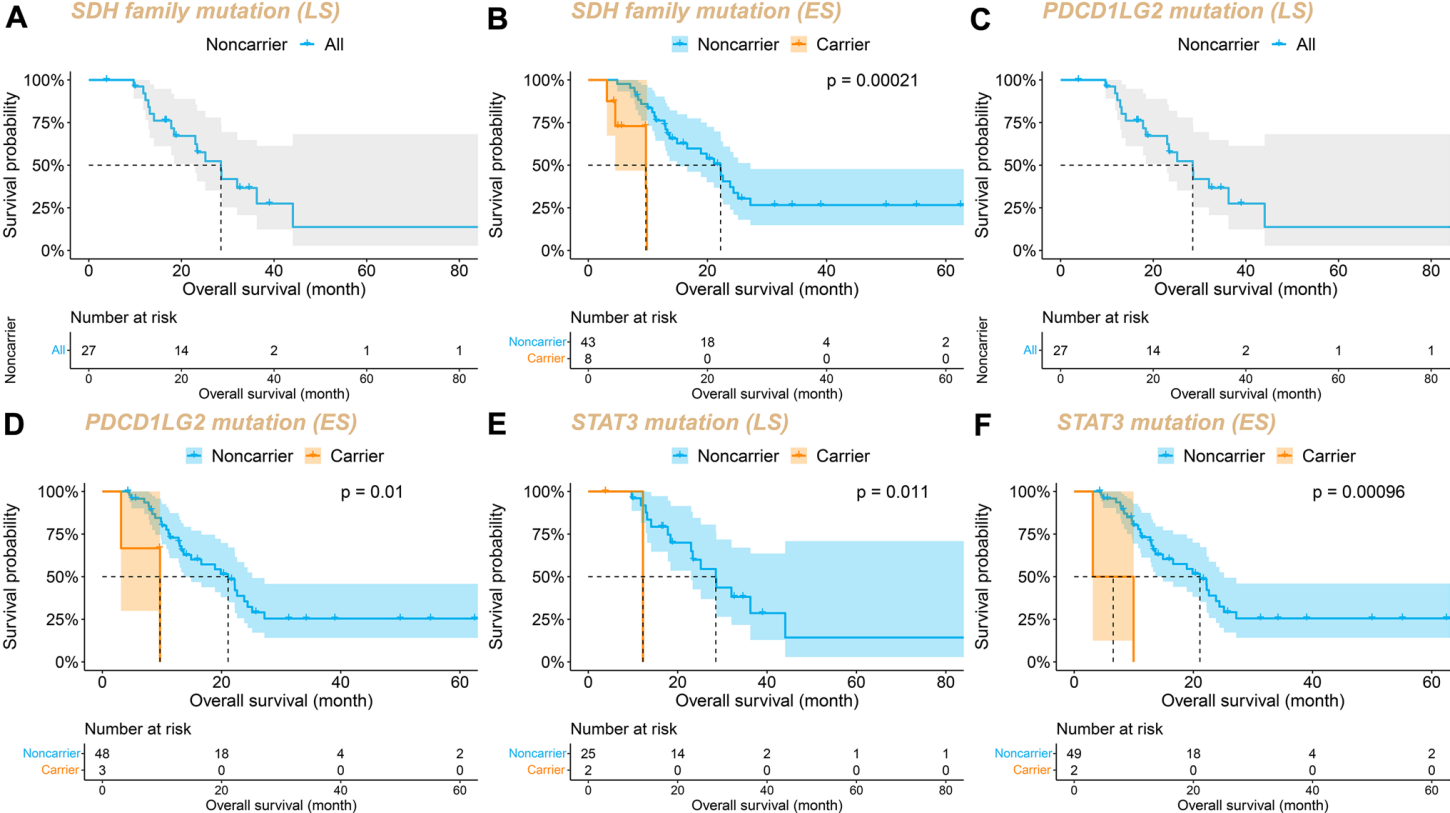
Kaplan–Meier curves for overall survival (OS) based on mutation status and tumor stage. **A** Impact of *SDH* family mutations on the prognosis of limited-stage (LS) patients, **B** impact of *SDH* family mutations on the prognosis of extensive-stage (ES) patients, **C** influence of *PDCD1LG2* mutations in LS cohort, **D** influence of *PDCD1LG2* mutations in ES cohort, **E** effect of *STAT3* mutations on LS cohort, and **F** effect of *STAT3* mutations on ES cohort

Importantly, to investigate whether patients’ staging would influence the predictive potential of these mutations on chemotherapy prognosis, we further divided the patient cohort into limited-stage (LS) and extensive-stage (ES) groups. The results demonstrated that in the LS group, which typically exhibits better chemotherapy outcomes, no patients carrying *SDHB/C/D* or *PDCD1LG2* mutations were observed (Fig. 5A and C). Conversely, in the ES group, patients with *SDHB/C/D* or *PDCD1LG2* mutations had significantly lower OS than non-carriers (Fig. 5B and D). Additionally, irrespective of the LS or ES group, *STAT3* gene mutation carriers exhibited significantly shorter OS than non-carriers (Fig. 5E and F), and LS patients with *AURKA* or *PMS2* mutations had poorer chemotherapy outcomes compared to non-carriers (Fig. S2A and S2C). No other statistically significant prognostic stratification differences were observed in either the LS or ES groups (Fig. S2), suggesting that the predictive value of these gene mutations for SCLC patient chemotherapy prognosis is fundamentally independent of tumor staging.

### Verification of the prognostic ability of SNVs

To verify our results, we utilized the cBioPortal, an open-source platform that enables interactive analysis of complex cancer genomics data sets. Our verification cohort comprised 239 SCLC patients. Upon analysis, we discovered that 6 of these patients from 3 study IDs carried 7 *SDH* family mutations (6 *SDHA* mutations and 1 *SDHB* mutation), 4 patients with 4 *STAT3* mutations, and no patients with *PDCD1LG2* mutation. Furthermore, our research indicated that SCLC patients with *SDH* family mutations frequently possess both *TP53* and *RB1* mutations (Table 2). Upon conducting further analysis of the association between these mutations and OS, we observed that individuals with *SDH* family mutations exhibited a relatively shorter median survival time, although the difference was not statistically significant (Fig. S3A). Conversely, no significant association was found between *STAT3* mutations and OS among patients (Fig. S3B).

**Table 2:** Clinical and genomic characteristics of SCLC patient’s harbored *SDH* and *STAT3* mutations in cbioportal database

Study ID	Patient ID	Diagnosis age	Sex	UICC tumor stage	Overall survival (months)	Overall survival status	*SDH* or *STAT3* mutation	*TP53* mutation	*RB1* mutation
sclc_ucologne_2015	sclc_ucologne_2015_S00938	58	Female	IV	15	1:DECEASED	*SDHA* A466V *SDHA* E473V	P151S	L477Mfs*17, D479Vfs*14
sclc_ucologne_2015	sclc_ucologne_2015_S01170	71	Male	Ia	20	1:DECEASED	*SDHB* G182W	G266E	–
sclc_ucologne_2015	sclc_ucologne_2015_S02279	58	Male	IIb	1	1:DECEASED	*SDHA* T298 =	V272L	K791*
sclc_ucologne_2015	sclc_ucologne_2015_S02273	58	Female	IV	24	1:DECEASED	*SDHA* A172P	E68Rfs*55	E817*
Small Cell Lung Cancer (Johns Hopkins, Nat Genet 2012)	134398	NA	NA	NA	NA	NA	*SDHA* A391S	P47Rfs*76	W75*
Small Cell Lung Cancer (CLCGP, Nat Genet 2012)	S00501	55	Female	NA	NA	NA	*SDHA* T656P *STAT3* A35V	R158G	Y454*
Small Cell Lung Cancer (U Cologne, Nature 2015)	sclc_ucologne_2015_S01563	53	Male	NA	NA	NA	*STAT3* V463Dfs*79	R196P	X738_splice
Small Cell Lung Cancer (U Cologne, Nature 2015)	sclc_ucologne_2015_S02287	62	Male	Ia	23	1:DECEASED	*STAT3* K363I	H179L	–
Small Cell Lung Cancer (Johns Hopkins, Nat Genet 2012)	585260	NA	NA	NA	NA	NA	*STAT3* M200I	–	–

*Termination mutation

^=^Synonymous mutation

### Molecular characteristics of prognostic model grouping

**Fig. 6:**
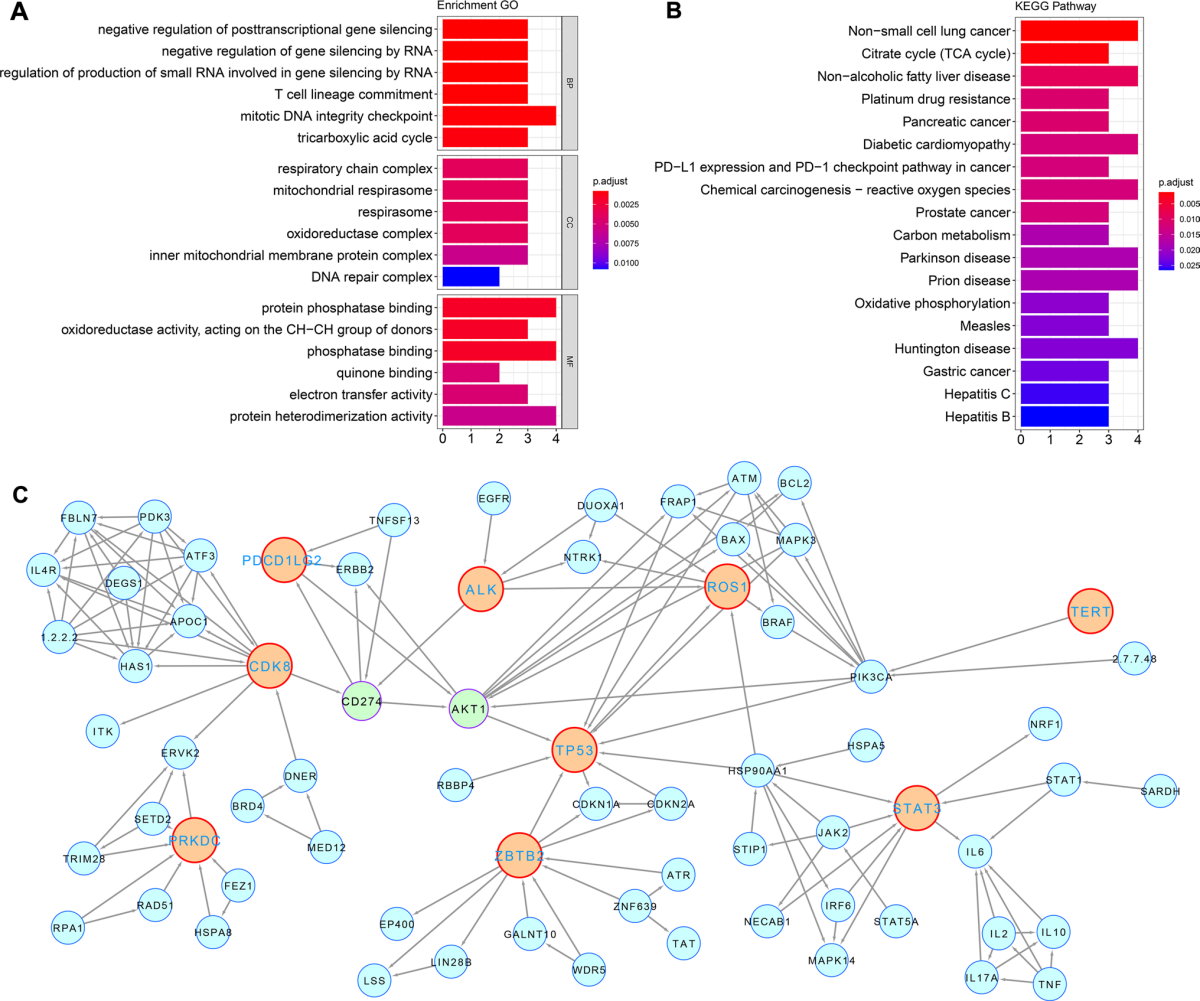
Bioinformatics analysis of prognostic-related mutated genes selected by random forest (RF) and support vector machines (SVM). **A** Gene ontology (GO) function enrichment of the top 20 genes selected in the prognostic model, **B** Kyoto Encyclopedia of Genes and Genomes (KEGG) pathway enrichment of the top 20 genes selected in the prognostic model, **C** the signaling regulatory networks of the top 20 genes selected in the prognostic model

To determine the possible pathways involving the genes included in the model, we conducted Gene Ontology (GO) functional annotation analysis and Kyoto Encyclopedia of Genes and Genomes (KEGG) pathway enrichment analysis for the 20 genes included in the model. The molecular function annotation revealed that the model included genes involved in the tricarboxylic acid cycle, mitochondrial oxidative phosphorylation, and other functions (Fig. 6A). Correspondingly, KEGG analysis demonstrated that genes involved in pathways were enriched in various tumor-related and metabolism-related pathways, including the tricarboxylic acid cycle, carbon metabolism, oxidative phosphorylation, platinum drug resistance, and PD-L1 expression (Fig. 6B). Simultaneously, KEGG & GO analysis results indicated that the target gene also participates in RNA post-transcriptional regulation and DNA damage repair.

To investigate the functional effects of the selected genes, we analyzed the gene-regulatory network of these genes in cancer, particularly in lung tumors. The results showed that among the 20 selected genes that contributed to the prognosis prediction of SCLC chemotherapy, 9 were located in the same signal regulatory network and mostly at key nodes, indicating that *CDK8*-*CD274*-*AKT1*-*TP53* may be an essential signal regulatory axis determining the prognosis of SCLC chemotherapy (Fig. 6C). Additionally, the regulatory network suggested that mutated genes were associated with cytokine regulation, such as *STAT3-IL-6*, indicating the potential correlation of the included genes in the model with the shaping of the SCLC immune microenvironment.

## Discussion

In this study, we explored 315 genes related to tumor cell cycle, angiogenesis, and DNA damage repair in 78 patients with unresectable LS-SCLC and ES-SCLC who received platinum-based dual-agent chemotherapy. The overall genes with high mutation frequency in the samples were consistent with previous literature reports, and the higher mutation levels observed in genes encoding histone-lysine N-methyltransferase 2 (KMT2) family proteins may be related to the race, disease stage of patients, and sample sources (surgery vs needle biopsy) [6, 28].

According to the random forest algorithm results, we selected the top 20 prognosis-related genes, including *SDHC*, *C11orf30*, *SDHB*, *PDCD1LG2*, *TOP2A*, *GRM3*, *SDHD*, *PRKDC*, *STAT3*, *AURKA*, etc. We used the support vector machine algorithm for final screening and model construction. The model’s area under the curve was 0.89, higher than the diagnostic ability based on disease stage and distant metastasis status. There were significant differences in PFS and OS between the two groups under the model. To date, this is the first study focusing on SCLC patients treated with standard first-line EC or EP and using machine learning algorithms to construct a prognostic model for SCLC.

The signaling regulatory networks revealed that the pathways genes were involved in were enriched in the tricarboxylic acid cycle, carbon metabolism, oxidative phosphorylation, platinum resistance, and PD-L1 expression (Fig. 6C), which corresponded with the molecular function annotation. *CDK8*-*CD274*-*AKT1*-*TP53* may be an important signaling axis determining the prognosis of SCLC chemotherapy, participating in the cytokine regulatory network, including IL-6. Furthermore, the KM curve analysis suggested that patients with *SDH* family gene mutations, *PDCD1LG2*, and *STAT3* gene mutations had worse prognoses after chemotherapy. All these characteristic analysis results suggest that the significantly shorter survival data in the poor prediction group in this study may be related to *SDH* complex dysfunction.

However, *SDH* is composed of six subunits, and deleterious mutations in any of them invariably result in functional destabilization of the entire complex [29]. *SDH* family mutations are considered to be associated with neuroendocrine tumors such as paraganglioma and pheochromocytoma [30, 31]. In the study of tumor-associated macrophages, the *SDH* complex participates in the metabolic reprogramming of melanoma by regulating the generation of mitochondrial reactive oxygen species, and the use of *SDH* inhibitors can inhibit the growth of melanoma [32]. Other types of tumorigenesis, including gastrointestinal stromal tumors (GISTs), renal, thyroid, melanoma, sarcoma, colon neuroblastoma [30, 33], pancreatic neuroendocrine tumors, Carney triad, and ganglioneuroma [34] have been shown to depend on *SDH* family mutations in some cases. The role of succinate accumulation in previously described tumors as an initiator in neoplasm invasion and metastasis has been well documented [35], suggesting the potential mechanism and importance of SDH in the highly invasive SCLC. This is the first study to find that SDH complex mutations may be related to the clinical significance, metabolic mechanism, and tumor development mechanism of SCLC. In the future, the mechanism of this gene mutation leading to tumor metabolism, occurrence, and development can be further explored in basic research, and the clinical value of predicting the efficacy of chemotherapy can be explored in clinical research prospectively.

In addition, the protein encoded by *PD-L2* and *PDCD1LG2* is one of the important components of the PD-1/PD-L1 and PD-L2 axis, which is involved in tumor immune escape [36]. Moreover, studies have shown that the expression level of PD-L2 is related to the efficacy of immunotherapy [37]. Based on these results, the prognosis model constructed in this study may be conducive to further screening of cohorts who benefit from immunotherapy combined with chemotherapy. This assumption requires follow-up retrospective and prospective clinical research.

Nevertheless, this study has several limitations. First, it is a multicenter retrospective study, and due to the relatively low prevalence of SCLC among lung cancer patients, the final sample size included is limited. Second, the study did not perform a detailed analysis of the impact of subsequent lines of treatment on patients' overall survival. Finally, the distribution of patients in the good prognosis cohort and the poor prognosis cohort within this study is uneven, which may introduce statistical bias. Therefore, further prospective multicenter studies involving a larger cohort should be undertaken to expand the understanding of our findings' ability to distinguish between SCLC patients with good and poor prognoses in response to chemotherapy. Additionally, the functional and molecular biological implications of these gene mutations warrant careful investigation in future research.

In summary, our study used machine learning algorithms to construct a prognostic model for SCLC patients treated with standard first-line EC or EP chemotherapy. The model showed potential in predicting clinical prognosis and identified several genes that may be involved in the development and progression of SCLC. Further exploration of the mechanisms by which these gene mutations contribute to tumor metabolism, occurrence, and development could provide valuable insights into the clinical value of predicting chemotherapy efficacy and identifying patients who may benefit from immunotherapy combined with chemotherapy.

## Supplementary Information

Additional file1 (DOCX 141 KB)

Additional file2 (DOCX 434 KB)

Additional file3 (DOCX 224 KB)

Additional file4 (DOCX 17 KB)

Additional file5 (DOCX 32 KB)

Additional file6 (DOCX 18 KB)

Additional file7 (DOCX 19 KB)

Additional file8 (DOCX 19 KB)

Additional file9 (DOCX 17 KB)

## Data Availability

The datasets generated and/or analysed during the current study are available from the corresponding authors on reasonable request.
