# Supplementary figures and images for: SDH mutations, as potential predictor of chemotherapy prognosis in small cell lung cancer patients
Source: Discov Oncol. 2023 Jun 5;14:89. doi: 10.1007/s12672-023-00685-4 (PMC10241767; doi:10.1007/s12672-023-00685-4)

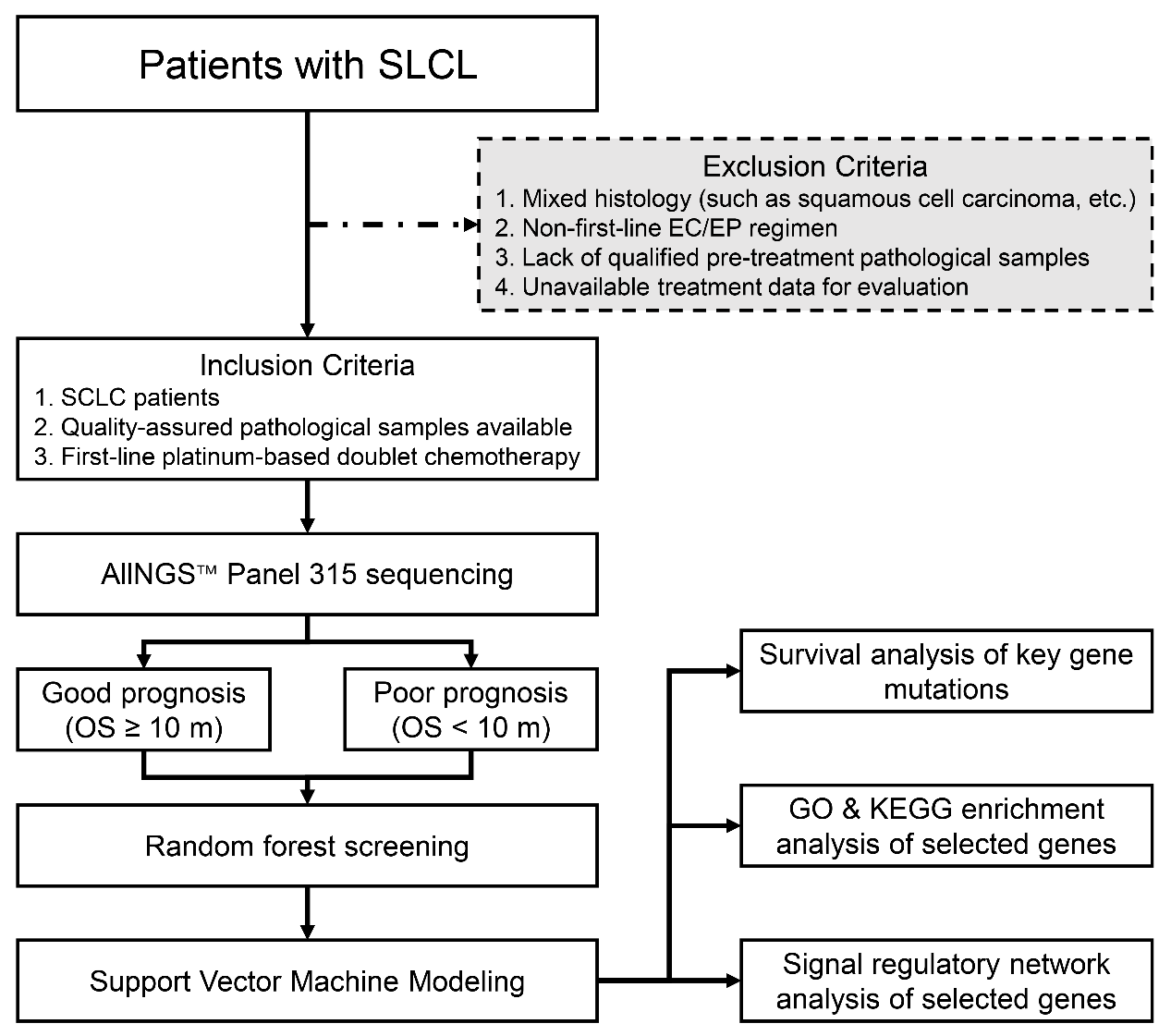


**Figure S1.** Study design and flowchart for constructing the prognostic model in SCLC patients.

Supplement: Supplementary file 1 — Additional file1 (DOCX 141 KB) [file 12672_2023_685_MOESM1_ESM.docx]
