# Supplementary material for: SDH mutations, as potential predictor of chemotherapy prognosis in small cell lung cancer patients
Source: Discov Oncol. 2023 Jun 5;14:89. doi: 10.1007/s12672-023-00685-4 (PMC10241767; doi:10.1007/s12672-023-00685-4)
Supplement: Supplementary file 2 — Additional file2 (DOCX 434 KB) [file 12672_2023_685_MOESM2_ESM.docx]

**
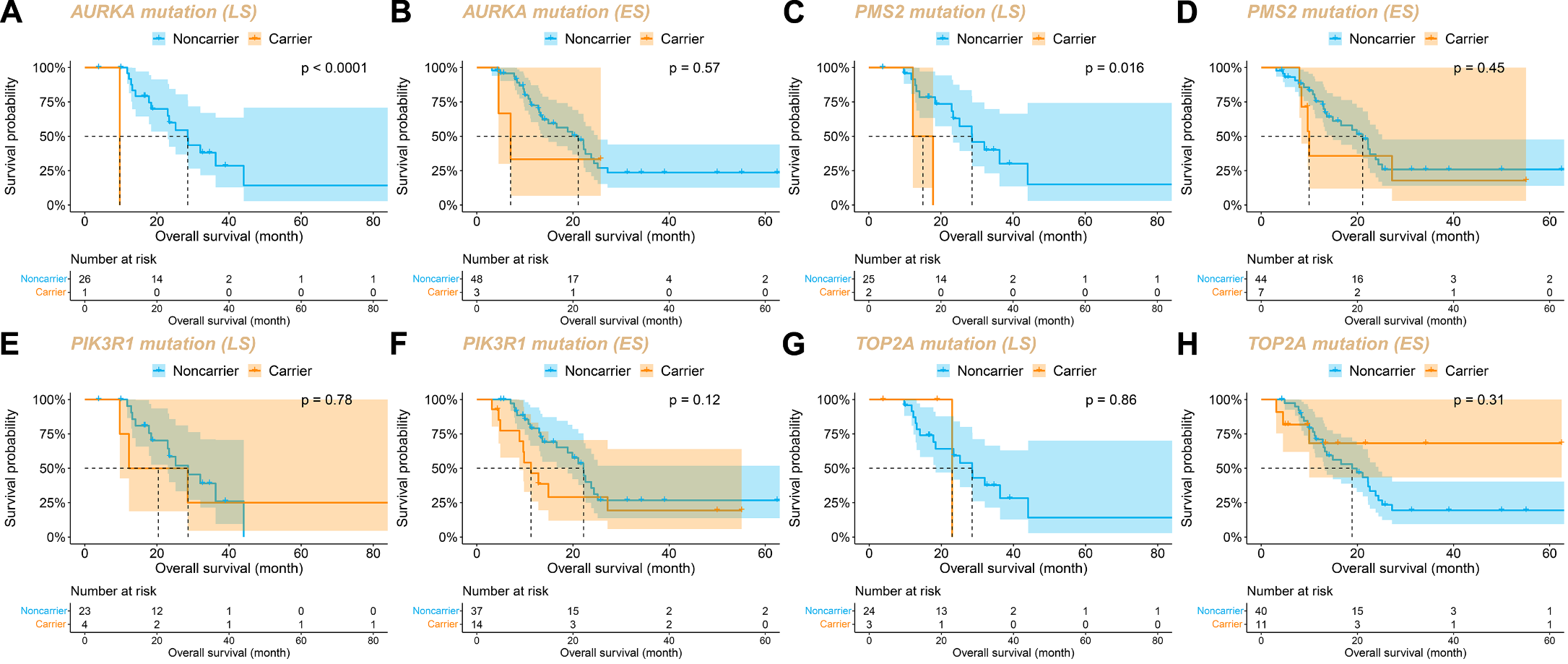
Figure S2.** Kaplan-Meier curves for overall survival (OS) based on mutation status and tumor stage. (A-B) Impact of *AURKA* mutations on the prognosis of (A) limited-stage (LS) patients and (B) extensive-stage (ES) patients, (C-D) Influence of *PMS2* mutations in (C) LS cohort and (D) ES cohort, (E-F) Effect of *PIK3R1* mutations on (E) LS cohort and (F) ES cohort, (G-H) Impact of *TOP2A* mutations on the prognosis of (G) LS patients and (H) ES patients.
