# Supplementary material for: SDH mutations, as potential predictor of chemotherapy prognosis in small cell lung cancer patients
Source: Discov Oncol. 2023 Jun 5;14:89. doi: 10.1007/s12672-023-00685-4 (PMC10241767; doi:10.1007/s12672-023-00685-4)
Supplement: Supplementary file 3 — Additional file3 (DOCX 224 KB) [file 12672_2023_685_MOESM3_ESM.docx]

**
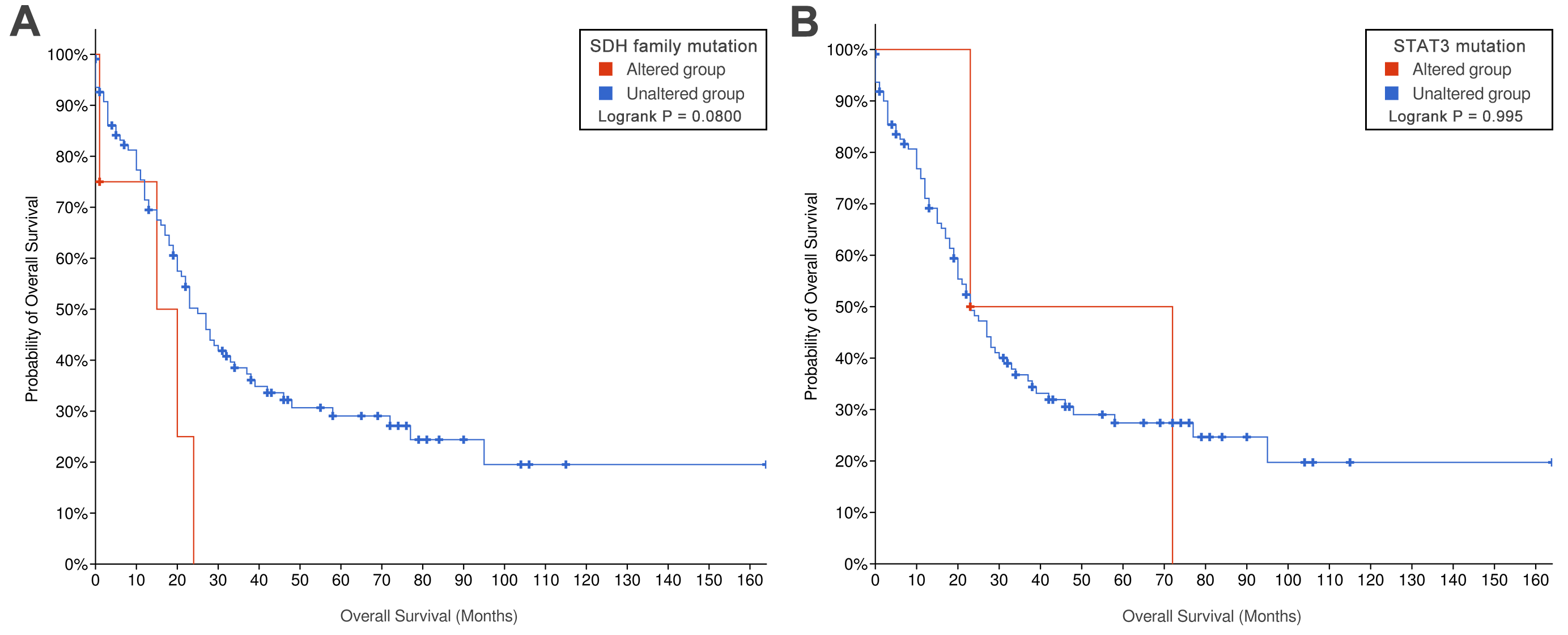
Figure S3.** Kaplan-Meier survival curves for overall survival (OS) based on the mutation status of (A) *SDH* family genes and (B) *STAT3*, as derived from the cBioPortal database.
