# Supplementary material for: SDH mutations, as potential predictor of chemotherapy prognosis in small cell lung cancer patients
Source: Discov Oncol. 2023 Jun 5;14:89. doi: 10.1007/s12672-023-00685-4 (PMC10241767; doi:10.1007/s12672-023-00685-4)
Supplement: Supplementary file 4 — Additional file4 (DOCX 17 KB) [file 12672_2023_685_MOESM4_ESM.docx]

**Supplementary Method**

***S1. Eligibility criteria***

1) Small cell lung cancer confirmed by histology/cytology.

2) Receive 4-6 cycles of etoposide combined with carboplatin or cisplatin as the first-line standard regimen recommended by CSCO guidelines.

3) Patients with sufficient samples of eligible SCLC tissues at baseline before first-line treatment.

4) Eastern Cooperative Oncology Group Performance Status (ECOG PS) score 0-2.

5) Baseline lesions and follow-up efficacy data were available.

***S2. Exclusion criteria***

1) Non-small cell lung cancer (including mixed types of small cell and non-small cell lung cancer).

2) Patients with other malignant tumors within 5 years before enrollment. Localized tumors that have been cured, such as basal cell carcinoma of the skin, squamous cell carcinoma of the skin, superficial bladder cancer, carcinoma in situ of the prostate, carcinoma in situ of the cervix, and carcinoma in situ of the breast can be enrolled.

3) Receive less than 2 cycles of standard first-line EC or EP. Patients receiving less than 2 cycles of standard first-line EC or EP due to disease progression (RECIST 1.1) (Eisenhauer et al., 2009) were eligible.

4) Sample quality before first-line treatment does not meet the basic requirements of the experiment (all cell blocks of the sample contain at least 20% tumor cells).

5) ECOG PS score >3.

**Reference**

Eisenhauer, E. A., Therasse, P., Bogaerts, J., Schwartz, L. H., Sargent, D., Ford, R., Dancey, J., Arbuck, S., Gwyther, S., Mooney, M., Rubinstein, L., Shankar, L., Dodd, L., Kaplan, R., Lacombe, D., & Verweij, J. (2009). New response evaluation criteria in solid tumours: revised RECIST guideline (version 1.1). *Eur J Cancer*, *45*(2), 228-247. https://doi.org/10.1016/j.ejca.2008.10.026
