# Supplementary material for: SDH mutations, as potential predictor of chemotherapy prognosis in small cell lung cancer patients
Source: Discov Oncol. 2023 Jun 5;14:89. doi: 10.1007/s12672-023-00685-4 (PMC10241767; doi:10.1007/s12672-023-00685-4)
Supplement: Supplementary file 5 — Additional file5 (DOCX 32 KB) [file 12672_2023_685_MOESM5_ESM.docx]

**Table S1.** Detailed information for each patient

| **ID** | **Age at diagnosis in years** | **Gender** | **Smoking** | **Two system** | **TNM classification** | **Distant metastasis** | **ECOG**  **PS** | **Chest Radiotherapy** | **Second-line treatment strategy** | **Posterior line immunotherapy** | **Survival**  **censored** | **Overall**  **survival**  **(days)** | **Prognosis** |
| --- | --- | --- | --- | --- | --- | --- | --- | --- | --- | --- | --- | --- | --- |
| 1 | 61 | Male | Present | Extensive | T2N3M1 | Bone, adrenal gland, liver | 1 | Yes | Chemotherapy | No | Yes | 754 | Good |
| 2 | 76 | Male | Present | Extensive | T4N3M1 | No | 1 | No | Chemotherapy | No | Yes | 731 | Good |
| 3 | 68 | Male | Present | Limited | T2N2M0 | No | 1 | Yes | Chemotherapy | No | Yes | 701 | Good |
| 4 | 51 | Male | Present | Extensive | T4N3M0 | No | 1 | No | Chemotherapy | No | Yes | 666 | Good |
| 5 | 40 | Male | Present | Extensive | T4N2M0 | No | 1 | Yes | Chemotherapy | No | No | 1652 | Good |
| 6 | 56 | Male | Never | Limited | T4N3M0 | No | 1 | Yes | Chemotherapy combined with radiotherapy | No | Yes | 395 | Good |
| 7 | 48 | Male | Present | Extensive | T4N3M1 | Brain, liver | 1 | Yes | Chemotherapy combined with radiotherapy | No | Yes | 409 | Good |
| 8 | 63 | Male | Past | Limited | T4N3M0 | No | 1 | Yes | Chemotherapy combined with radiotherapy | No | Yes | 690 | Good |
| 9 | 66 | Male | Past | Limited | T4N0M0 | No | 1 | No | Unknown | No | Yes | 367 | Good |
| 10 | 64 | Male | Present | Extensive | T2N1M1 | Bone | 2 | No | Anti-angiogenesis therapy | No | No | 1170 | Good |
| 11 | 68 | Male | Present | Limited | T3N2M0 | No | 0 | Yes | No | No | Yes | 534 | Good |
| 12 | 53 | Male | Present | Limited | T3N2M0 | No | 1 | Yes | Chemotherapy combined with immunotherapy | Yes | Yes | 961 | Good |
| 13 | 60 | Male | Past | Extensive | T4N3M1 | Brain | 0 | Yes | Chemotherapy | Yes | Yes | 498 | Good |
| 14 | 69 | Male | Past | Extensive | T2NxM1 | Brain | 1 | Yes | Unknown | No | Yes | 714 | Good |
| 15 | 65 | Male | Never | Limited | T2N3M0 | No | 1 | No | Chemotherapy | No | Yes | 856 | Good |
| 16 | 62 | Male | Never | Limited | T2N2M0 | Brain | 1 | Yes | Chemotherapy combined with radiotherapy | No | No | 1038 | Good |
| 17 | 63 | Male | Present | Extensive | T4N3M1 | Bone | 1 | No | Chemotherapy | No | Yes | 667 | Good |
| 18 | 60 | Male | Present | Limited | T2N1M0 | No | 0 | Yes | No | No | No | 979 | Good |
| 19 | 63 | Male | Past | Limited | T1N0M0 | No | 0 | No | Unknown | No | No | 1169 | Good |
| 20 | 65 | Male | Past | Limited | T1N2M0 | No | 1 | Yes | Chemotherapy combined with anti-vascular therapy | No | No | 300 | Good |
| 21 | 57 | Male | Present | Limited | T3N0M0 | No | 1 | No | Chemotherapy combined with immunotherapy | Yes | No | 1038 | Good |
| 22 | 72 | Female | Never | Extensive | T4N2M1 | Bone | 1 | No | Chemotherapy | No | Yes | 567 | Good |
| 23 | 77 | Male | Present | Extensive | T2N3M1 | Bone | 1 | Yes | Chemotherapy | No | Yes | 596 | Good |
| 24 | 74 | Female | Never | Extensive | T4N2M1 | No | 1 | Yes | Chemotherapy combined with radiotherapy | No | Yes | 384 | Good |
| 25 | 51 | Male | Never | Extensive | T3N3M1 | No | 1 | No | Chemotherapy | No | Yes | 633 | Good |
| 26 | 66 | Male | Present | Limited | T1N2M0 | No | 1 | Yes | No | No | Yes | 386 | Good |
| 27 | 66 | Male | Present | Extensive | T3N3M0 | No | 1 | No | Chemotherapy | No | Yes | 333 | Good |
| 28 | 59 | Female | Never | Limited | T3N0M0 | No | 1 | Yes | Chemotherapy | No | Yes | 552 | Good |
| 29 | 61 | Male | Past | Limited | T4N3M0 | No | 1 | Yes | Chemotherapy | No | Yes | 1088 | Good |
| 30 | 70 | Male | Present | Limited | T4N3M1 | No | 1 | Yes | Chemotherapy | No | Yes | 859 | Good |
| 31 | 59 | Male | Present | Extensive | TxNxM1 | Brain | 1 | No | Chemotherapy | No | No | 1978 | Good |
| 32 | 65 | Male | Never | Extensive | T4N1M1 | Adrenal gland | 1 | No | Unknown | No | Yes | 397 | Good |
| 33 | 27 | Male | Never | Limited | T2N2M0 | No | 1 | Yes | Chemotherapy | Yes | Yes | 1322 | Good |
| 34 | 72 | Male | Never | Extensive | T2N1M1 | Brain, adrenal gland | 1 | Yes | Chemotherapy | No | No | 384 | Good |
| 35 | 64 | Male | Present | Extensive | TxNxM1 | Brain | 1 | Yes | Chemotherapy combined with radiotherapy | No | No | 1499 | Good |
| 36 | 54 | Male | Present | Limited | T4N3M0 | No | 1 | Yes | Anti-angiogenesis therapy | No | Yes | 353 | Good |
| 37 | 61 | Male | Past | Extensive | T4N3M0 | No | 1 | Yes | Unknown | No | No | 401 | Good |
| 38 | 61 | Male | Present | Extensive | T4N3M1 | Bone | 1 | No | Anti-angiogenesis therapy | No | Yes | 447 | Good |
| 39 | 65 | Male | Never | Extensive | T4N2M1 | No | 1 | No | Chemotherapy combined with radiotherapy | No | No | 938 | Good |
| 40 | 55 | Male | Present | Extensive | T4N3M1 | Bone | 1 | Yes | Chemotherapy | Yes | No | 773 | Good |
| 41 | 58 | Male | Past | Extensive | T4N3M1 | Adrenal gland, liver | 1 | Yes | No | No | Yes | 338 | Good |
| 42 | 55 | Male | Never | Limited | T2N2M0 | No | 1 | No | Chemotherapy | No | Yes | 422 | Good |
| 43 | 80 | Male | Past | Extensive | T4N2M1 | Brain, bone | 1 | Yes | Chemotherapy | No | No | 478 | Good |
| 44 | 61 | Male | Present | Limited | T4N2M0 | No | 1 | Yes | Unknown | No | No | 565 | Good |
| 45 | 63 | Male | Never | Extensive | T1N0M1 | Brain, adrenal gland, bone | 1 | Yes | Chemotherapy combined with radiotherapy | No | Yes | 816 | Good |
| 46 | 61 | Male | Present | Extensive | T3N3M1 | Brain | 1 | Yes | Chemotherapy combined with radiotherapy | No | Yes | 678 | Good |
| 47 | 69 | Male | Never | Limited | T4N3M0 | No | 0 | Yes | Chemotherapy | No | No | 493 | Good |
| 48 | 67 | Male | Past | Extensive | T4N2M1 | No | 1 | No | Chemotherapy combined with anti-vascular therapy | No | No | 1026 | Good |
| 49 | 63 | Male | Past | Extensive | T4N3M1 | Liver | 1 | No | No | No | Yes | 322 | Good |
| 50 | 53 | Male | Past | Extensive | T4N1N1 | Bone | 1 | Yes | No | No | No | 1874 | Good |
| 51 | 65 | Male | Present | Limited | T1N2M0 | No | 1 | Yes | Unknown | No | No | 502 | Good |
| 52 | 56 | Male | Present | Limited | T4N2M0 | No | 1 | Yes | Chemotherapy combined with immunotherapy and radiotherapy | Yes | Yes | 755 | Good |
| 53 | 67 | Male | Present | Extensive | T1N2M1 | Brain | 0 | Yes | Chemotherapy | No | No | 650 | Good |
| 54 | 78 | Female | Never | Limited | T2N3M0 | No | 1 | No | Chemotherapy combined with immunotherapy | No | No | 705 | Good |
| 55 | 48 | Male | Present | Extensive | T2N2M1 | Brain | 1 | No | Unknown | No | No | 301 | Good |
| 56 | 71 | Male | Present | Extensive | T4N3M1 | No | 1 | Yes | Unknown | No | No | 423 | Good |
| 57 | 80 | Male | Present | Limited | T2N1M0 | No | 1 | No | Chemotherapy | No | No | 2608 | Good |
| 58 | 72 | Male | Never | Extensive | T2N1M1 | Liver | 1 | No | Chemotherapy combined with radiotherapy | No | No | 343 | Good |
| 59 | 75 | Male | Never | Extensive | T4N2M1 | Brain | 1 | Yes | Chemotherapy combined with immunotherapy | Yes | No | 610 | Good |
| 60 | 69 | Male | Present | Extensive | T4N3M1 | No | 2 | No | No | No | Yes | 389 | Good |
| 61 | 61 | Male | Present | Extensive | T1N3M1 | Bone, adrenal gland | 1 | No | Chemotherapy | No | No | 167 | Poor |
| 62 | 66 | Male | Past | Limited | T3N0M0 | No | 1 | Yes | No | No | Yes | 291 | Poor |
| 63 | 63 | Male | Never | Extensive | T2N3M1 | Liver | 1 | No | Chemotherapy | No | Yes | 251 | Poor |
| 64 | 75 | Male | Present | Extensive | T4N2M0 | No | 0 | Yes | No | No | Yes | 233 | Poor |
| 65 | 50 | Male | Present | Limited | T2N2M0 | No | 1 | No | Unknown | No | No | 115 | Poor |
| 66 | 63 | Male | Past | Extensive | T4N3M1 | Bone, liver | 2 | No | Chemotherapy | No | No | 128 | Poor |
| 67 | 53 | Male | Present | Extensive | T4N2M1 | Bone | 3 | Yes | Unknown | No | Yes | 211 | Poor |
| 68 | 61 | Male | Present | Extensive | T4N3M1 | Brain, bone | 1 | No | No | No | Yes | 146 | Poor |
| 69 | 66 | Male | Present | Extensive | T4N3M1 | No | 1 | No | Unknown | No | No | 149 | Poor |
| 70 | 57 | Male | Present | Extensive | T4N3M0 | No | 1 | Yes | Chemotherapy | No | No | 247 | Poor |
| 71 | 73 | Male | Present | Extensive | T4NxM1 | Brain | 1 | No | Unknown | No | Yes | 295 | Poor |
| 72 | 58 | Male | Present | Extensive | T4N2M0 | No | 0 | No | Chemotherapy | No | Yes | 290 | Poor |
| 73 | 78 | Male | Past | Extensive | T3N2M0 | No | 2 | No | Chemotherapy | No | Yes | 136 | Poor |
| 74 | 61 | Male | Present | Extensive | T4N3M1 | Liver | 1 | No | No | No | Yes | 94 | Poor |
| 75 | 62 | Male | Present | Extensive | T3N3M1 | Liver | 1 | Yes | Chemotherapy | No | Yes | 238 | Poor |
| 76 | 71 | Female | Never | Extensive | T2N0M1 | Bone, liver | 0 | Yes | Anti-angiogenesis therapy | No | No | 287 | Poor |
| 77 | 50 | Male | Present | Extensive | T4N2M1 | Bone | 0 | No | Chemotherapy combined with radiotherapy | No | Yes | 266 | Poor |
| 78 | 74 | Male | Past | Extensive | T2N2Mx | No | 1 | No | No | No | Yes | 299 | Poor |
