# Supplementary material for: SDH mutations, as potential predictor of chemotherapy prognosis in small cell lung cancer patients
Source: Discov Oncol. 2023 Jun 5;14:89. doi: 10.1007/s12672-023-00685-4 (PMC10241767; doi:10.1007/s12672-023-00685-4)
Supplement: Supplementary file 6 — Additional file6 (DOCX 18 KB) [file 12672_2023_685_MOESM6_ESM.docx]

**Table S2.** Subgroup analysis of correlation between PFS and clinical characteristics of patients with advanced SCLC receiving platinum containing dual drug chemotherapy in the first line.

| **Predictors** | **Single factor regression** | | **Multifactor regression** | |
| --- | --- | --- | --- | --- |
|  | **HR ^b.^, 95% CI ^c.^** | ***P* *value*** | **HR ^b.^, 95% CI ^c.^** | ***P* *value**** |
| **Age** | | | | |
| *＜65 years old* | 1.000 |  |  |  |
| *≥65 years old* | 1.068 (0.643-1.773) | 0.801 |  |  |
| **Gender** | | | | |
| *Female* | 1.000 |  | 1.000 |  |
| *Male* | 0.378 (0.148-0.962) | **0.041^* d.^** | 0.576 (0.213-1.555) | 0.276 |
| **Staging** | | | | |
| *Limited-stage* | 1.000 |  |  |  |
| *Extensive-stage* | 1.662 (0.979-2.821) | 0.060 |  |  |
| **Smoking** | | | | |
| *Never* | 1.000 |  |  |  |
| *Present/Past* | 0.790 (0.453-1.378) | 0.406 |  |  |
| **ECOG PS ^a.^** | | | | |
| *0-1* | 1.000 |  |  |  |
| *2-3* | 2.212 (0.796-6.150) | 0.128 |  |  |
| **Distant metastasis** | | | | |
| *No* | 1.000 |  |  |  |
| *Yes* | 1.303 (0.799-2.126) | 0.289 |  |  |
| **Radiotherapy** | | | | |
| *No* | 1.000 |  |  |  |
| *Yes* | 0.802 (0.486-1.324) | 0.388 |  |  |
| **Predicting Group** | | | | |
| *0* | 1.000 |  | 1.000 |  |
| *1* | 3.169 (1.598-6.282) | **0.001^* d.^** | 2.820 (1.371-5.801) | **0.005^* d.^** |

Notes:

^a.^ ECOG PS, Eastern Cooperative Oncology Group Performance Status.

^b.^ HR, Hazard ratio.

^c.^ CI, confidence interval.

^d.^ Bold value, statistically significant; *, at the level of P<0.050.
