# Supplementary material for: SDH mutations, as potential predictor of chemotherapy prognosis in small cell lung cancer patients
Source: Discov Oncol. 2023 Jun 5;14:89. doi: 10.1007/s12672-023-00685-4 (PMC10241767; doi:10.1007/s12672-023-00685-4)
Supplement: Supplementary file 7 — Additional file7 (DOCX 19 KB) [file 12672_2023_685_MOESM7_ESM.docx]

**Table S3.** Subgroup analysis of correlation between OS and clinical characteristics of patients with advanced SCLC receiving platinum containing dual drug chemotherapy in the first line.

| **Predictors** | **Single factor regression** | | **Multifactor regression** | |
| --- | --- | --- | --- | --- |
|  | **HR ^b.^, 95% CI ^c.^** | ***P* *value*** | **HR ^b.^, 95% CI ^c.^** | ***P* *value*** |
| **Age** | | | | |
| *＜65 years old* | 1.000 |  |  |  |
| *≥65 years old* | 1.186 (0.652-2.157) | 0.579 |  |  |
| **Gender** | | | | |
| *Female* | 1.000 |  |  |  |
| *Male* | 1.360 (0.416-4.447) | 0.611 |  |  |
| **Staging** | | | | |
| *Limited-stage* | 1.000 |  |  |  |
| *Extensive-stage* | 1.523 (0.826-2.806) | 0.178 |  |  |
| **Smoking** | | | | |
| *Never* | 1.000 |  |  |  |
| *Present/Past* | 1.080 (0.547-2.133) | 0.824 |  |  |
| **ECOG PS ^a.^** | | | | |
| *0-1* | 1.000 |  |  |  |
| *2-3* | 1.763 (0.543-5.724) | 0.345 |  |  |
| **Distant metastasis** | | | | |
| *No* | 1.000 |  |  |  |
| *Yes* | 1.141 (0.636-2.048) | 0.657 |  |  |
| **Radiotherapy** | | | | |
| *No* | 1.000 |  |  |  |
| *Yes* | 0.768 (0.430-1.374) | 0.374 |  |  |
| **Back-line immunotherapy** | | | | |
| *No* | 1.000 |  |  |  |
| *Yes* | 0.530 (0.189-1.483) | 0.226 |  |  |
| **Predicting Group** | | | | |
| *0* | 1.000 |  | 1.000 |  |
| *1* | 2.512 (1.107-5.701) | **0.028^* d.^** | 2.512 (1.107-5.701) | **0.028^* d.^** |

Notes:

^a.^ ECOG PS, Eastern Cooperative Oncology Group Performance Status.

^b.^ HR, Hazard ratio.

^c.^ CI, confidence interval.

^d.^ Bold value, statistically significant; *, at the level of P <0.050.
