# Supplementary material for: SDH mutations, as potential predictor of chemotherapy prognosis in small cell lung cancer patients
Source: Discov Oncol. 2023 Jun 5;14:89. doi: 10.1007/s12672-023-00685-4 (PMC10241767; doi:10.1007/s12672-023-00685-4)
Supplement: Supplementary file 8 — Additional file8 (DOCX 19 KB) [file 12672_2023_685_MOESM8_ESM.docx]

**Table S4.** Distribution of the top 20 RF-screened genes between the two prognosis cohorts.

| Gene | Mutation frequency | | Poisson *P value* | Odds ratio |
| --- | --- | --- | --- | --- |
|  | Good (*n* = 60) | Poor (*n* = 18) |  |  |
| *TP53* | 33 (55.0%) | 9 (50.0%) | 1.000 | 0.909 |
| *PRKDC* | 31 (51.7%) | 7 (38.9%) | 0.569 | 0.753 |
| *ROS1* | 26 (43.3%) | 4 (22.2%) | 0.279 | 0.513 |
| *ALK* | 21 (35.0%) | 8 (44.4%) | 0.516 | 1.270 |
| *KDM6A* | 15 (25.0%) | 9 (50.0%) | 0.141 | 2.000 |
| *GRM3* | 13 (21.7%) | 9 (50.0%) | 0.071 | 2.308 |
| *PIK3R1* | 10 (16.7%) | 8 (44.4%) | **0.046^* b.^** | 2.667 |
| *C11orf30* | 9 (15.0%) | 7 (38.9%) | 0.070 | 2.593 |
| *TOP2A* | 7 (11.7%) | 7 (38.9%) | **0.025^* b.^** | 3.333 |
| *ERG* | 8 (13.3%) | 5 (27.8%) | 0.193 | 2.083 |
| *ZBTB2* | 8 (13.3%) | 5 (27.8%) | 0.193 | 2.083 |
| *PMS2* | 4 (6.7%) | 5 (27.8%) | **0.035^* b.^** | 4.167 |
| *TERT* | 5 (8.3%) | 2 (11.1%) | 0.665 | 1.333 |
| *STAT3* | 1 (1.7%) | 3 (16.7%) | **0.041^* b.^** | 10.000 |
| *AURKA* | 1 (1.7%) | 3 (16.7%) | **0.041^* b.^** | 10.000 |
| *CDK8* | 1 (1.7%) | 2 (11.1%) | 0.135 | 6.667 |
| *SDHC* | 0 (0.0%) | 3 (16.7%) | **0.012^* b.^** | Inf ^a.^ |
| *SDHB* | 0 (0.0%) | 3 (16.7%) | **0.012^* b.^** | Inf ^a.^ |
| *SDHD* | 0 (0.0%) | 3 (16.7%) | **0.012^* b.^** | Inf ^a.^ |
| *PDCD1LG2* | 0 (0.0%) | 3 (16.7%) | **0.012^* b.^** | Inf ^a.^ |

Notes:

^a.^ Inf, Infinite.

^b.^ Bold value, statistically significant; *, at the level of P <0.050.
