# Supplementary material for: SDH mutations, as potential predictor of chemotherapy prognosis in small cell lung cancer patients
Source: Discov Oncol. 2023 Jun 5;14:89. doi: 10.1007/s12672-023-00685-4 (PMC10241767; doi:10.1007/s12672-023-00685-4)
Supplement: Supplementary file 9 — Additional file9 (DOCX 17 KB) [file 12672_2023_685_MOESM9_ESM.docx]

**Table S5.** Distribution of the *MYC* family, *KMT2D* and *PIK3CA* genes between the two prognosis cohorts.

| Gene | Mutation frequency | | Poisson *P value* | Odds ratio |
| --- | --- | --- | --- | --- |
|  | Good (*n* = 60) | Poor (*n* = 18) |  |  |
| *MYC* | 3 (5.0%) | 1 (5.6%) | 1.000 | 1.111 |
| *MYCL* | 1 (1.7%) | 0 (0.0%) | 1.000 | 0.000 |
| *KMT2D* | 35 (58.3%) | 12 (66.7%) | 0.729 | 1.143 |
| *PIK3CA* | 9 (15.0%) | 1 (5.6%) | 0.471 | 0.370^.^ |
